# 3D-printed wound dressing platform for protein administration based on alginate and zinc oxide tetrapods

**DOI:** 10.1186/s40580-023-00401-6

**Published:** 2023-11-16

**Authors:** Philipp Schadte, Franziska Rademacher, Gerrit Andresen, Marie Hellfritzsch, Haoyi Qiu, Gregor Maschkowitz, Regine Gläser, Nina Heinemann, Daniel Drücke, Helmut Fickenscher, Regina Scherließ, Jürgen Harder, Rainer Adelung, Leonard Siebert

**Affiliations:** 1 Functional Nanomaterials, Department for Material Science, Kiel University, Kiel, Germany; 2 Department of Dermatology, Kiel University and University Medical Center Schleswig-Holstein, Kiel, Germany; 3 Institute for Infection Medicine, Kiel University and University Medical Center Schleswig-Holstein, Kiel, Germany; 4 Department of Pharmaceutics and Biopharmaceutics, Kiel University, Kiel, Germany; 5 Department of Reconstructive Surgery, Kiel University and University Medical Center Schleswig-Holstein, Kiel, Germany; 6 Kiel Nano, Surface and Interface Science - KiNSIS, Kiel University, Kiel, Germany

**Keywords:** Nanomaterials, Biofabrication, Wound healing, Protein decoration, Dermatology, Antibacterial activity, Zinc oxide tetrapods, 3D microcrystals

## Abstract

Wound treatment requires a plethora of independent properties. Hydration, anti-bacterial properties, oxygenation and patient-specific drug delivery all contribute to the best possible wound healing. Three-dimensional (3D) printing has emerged as a set of techniques to realize individually adapted wound dressings with open porous structure from biomedically optimized materials. To include all the desired properties into the so-called bioinks is still challenging. In this work, a bioink system based on anti-bacterial zinc oxide tetrapods (t-ZnO) and biocompatible sodium alginate is presented. Additive manufacturing of these hydrogels with high t-ZnO content (up to 15 wt.%) could be realized. Additionally, protein adsorption on the t-ZnO particles was evaluated to test their suitability as carriers for active pharmaceutical ingredients (APIs). Open porous and closed cell printed wound dressings were tested for their cell and skin compatibility and anti-bacterial properties. In these categories, the open porous constructs exhibited protruding t-ZnO arms and proved to be anti-bacterial. Dermatological tests on ex vivo skin showed no negative influence of the alginate wound dressing on the skin, making this bioink an ideal carrier and evaluation platform for APIs in wound treatment and healing.

**Supplementary Information:**

The online version contains supplementary material available at 10.1186/s40580-023-00401-6.

## Introduction

Complex wounds from burns or surgery are difficult to treat and thus display a major burden to affected patients and the health care system in general [1, 2]. The difficulty lies in the multi-factorial causes of the wound and the individuality of the size, depth and degree of infection per patient. While one wound may be infected with a certain type of multi resistant microorganisms, another wound is too dry and yet another lacks the means for revascularization. Most often it is a case-specific combination of these issues that require individual and complex treatments.

Smart interventions with multi-functional wound dressings have therefore become an attractive clinical research topic. Especially the manufacturing by additive manufacturing, i.e., three-dimensional (3D) printing has been demonstrated to offer individual treatment options by the design of versatile bioinks that can address many of the necessary aspects for effective wound healing. Closed structures are commonly used to protect the wound from direct contamination or pathogen exposure. An open porous structure on the other hand can provide oxygenation, and a 3D-design that fits the wounds perfectly is very promising [3, 4].

By incorporating functional particles into the bioink formulation, the enhancement of healing capabilities can be achieved. Zinc oxide, due to its antibacterial properties, has garnered significant interest for application in wound dressings, especially in nanoparticle (NP) form [5–7]. These particles can be readily integrated into bioinks and are therefore investigated extensively in various material combinations. However, NPs not only impact bacterial proliferation but also affect the surrounding healthy tissue, making them less biocompatible compared to other ZnO variants. In the form of tetrapodal microcrystals, ZnO retains remarkable antibacterial properties while exhibiting lower toxicity towards surrounding tissue [8]. Furthermore, its three-dimensional structure offers additional advantages over conventional NPs. The individual arms of tetrapods can protrude from the bioink surface, establishing direct contact between ZnO and the targeted bacteria.

The research activities in this field are numerous and include smart, triggerable wound dressings, hybrid synthetic and natural hydrogel systems and drug delivery systems based on 3D printed soft materials to address the needs of complex wounds [9–16]. In this work, we propose a multi-functional bioink platform based on sodium alginate hydrogel and nanostructured tetrapodal zinc oxide (t-ZnO), that can be functionalized with active pharmaceutical ingredients (APIs) Additional file 1: wound healing. For this bioink we demonstrate its printability and test the cell compatibility and anti-bacterial properties of open- and closed cell constructs containing varying amounts of t-ZnO.

**Fig. 1:**
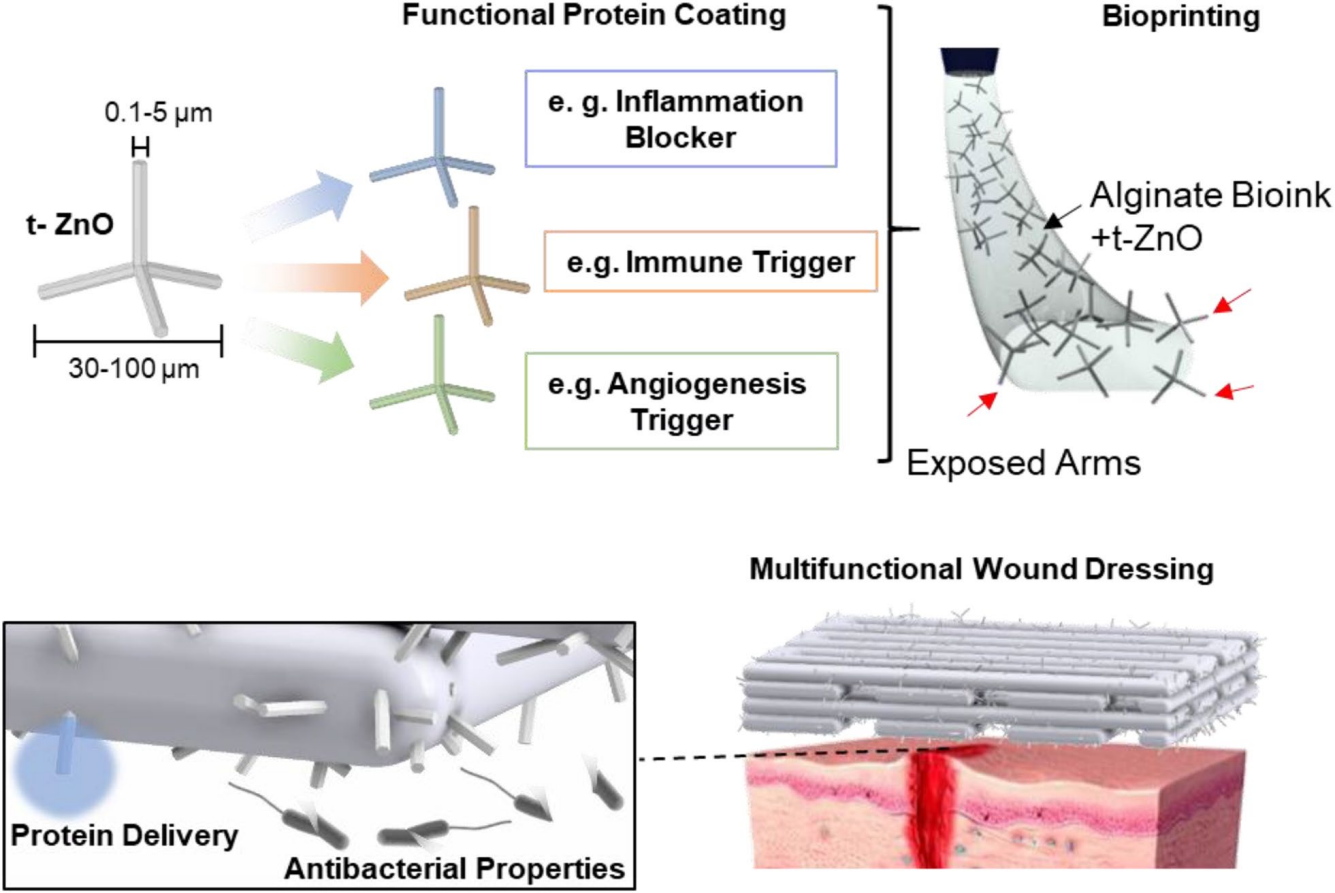
Workflow for alginate-based inks: Tetrapodal zinc oxide, produced via the flame transport synthesis, can be decorated with cytokines for advanced biomedical functionality. Depending on the specific wound requirements functions like immune triggering, inflammation blocking or angiogenesis supporters. These cytokine-laden t-ZnO particles can then be embedded into sodium alginate solution and bioprinted. Due to the geometry of the tetrapods, they tend to protrude from the surface of the hydrogel, forming a direct contact with the surrounding. Via bioprinting, open porous constructs can be built, based on the requirements of the specific wound to be treated. The protruding tetrapodal arms can then release the decorated cytokines and additionally act as antibacterial agents. The alginate-based ink covers the wound and protects the surface from contamination while still facilitating oxygen permeation

The core principle is demonstrated in Fig. 1.

## Experimental methods

### Tetrapodal ZnO synthesis

Tetrapodal zinc oxide particles were synthesized via the flame transport synthesis described in previous works [17–20]. In brief, polyvinyl butyral (PVB) powder supplied by Kuraray Europe GmbH was mixed with zinc microparticles (particle size < 5 µm, art.no. GF47693122, Sigma Aldrich, Germany) in a 2:1 ratio by weight. This mixture of powders was put into an alumina crucible which was placed into a pre-heated furnace at 900 °C for 30 min. The zinc was protected from oxidation by the PVB which reacted with the furnace’s oxygen to form CO_2_. When the mixture had reached approximately 900 °C, much of the zinc evaporated and started to react with incoming oxygen once the PVB has been completely oxidized to CO_2_. From the vapor phase, small zinc oxide nuclei were formed which developed to tetrapods over the course of 30 min. After this duration, the crucible was filled with a white, fluffy powder consisting of individual zinc oxide tetrapods. These were harvested into separate containers and could be used for further experimentation.

### Bioink fabrication

The carrier hydrogel for printing the t-ZnO-laden constructs was chosen to be an aqueous solution of sodium alginate (from here on just called alginate) because of its excellent properties such as biocompatibility, facile gelation and the capability for derivation. [21, 22]. Aside from those, alginate offers high printability and has thus been explored in many works about biofabrication. Powdered sodium alginate in food grade was obtained from Dragonspice Naturwaren (art.no.: alginate) [23]. A high concentration of alginate in water at approximately 20 wt.% yields a malleable, dough-like paste, that is a very good basis for bioprinting. It exhibits both shear-thinning behavior as well as viscoelastic properties leading to shape retention after extrusion. Adding t-ZnO was done, by mixing the alginate and the t-ZnO powders gently and then adding water in the desired amount. Everything was mixed by stirring with hand for about one min. The alginate-based inks were left for gelation overnight. Solutions with concentrations of 0 wt.%, 5 wt.% and 15 wt.% of t-ZnO have been chosen for bioprinting and further biological testing. These concentrations stem from prior research, demonstrating that tetrapods exhibit approximately 100 times lower toxicity than ZnO nanoparticles at a 5 wt.% concentration [8]. Furthermore, Siebert et al. [9] have established that even concentrations as low as 0.5 wt.% yield a significant antibacterial effect. In addition to these findings, the selected concentrations were carefully chosen to guarantee a sufficient number of tetrapod arms protruding from the surface of the bioinks. A concentration of 0 wt.% acted as the reference to observe the effect of the pure alginate gel.

### 3D-printing

After fabrication, the bioinks were processed with 3D-printing setup capable of extruding highly viscous pastes like the ZnO loaded hydrogels. The custom-made setup consists of a moveable xy-stage with a polytetrafluroethylene (PTFE) printbed and a hydraulic extrusion unit connected to the z axis of the printer. This hydraulic system enables the application of high pressure to the bioinks and therefore allows the use of nozzles with orifices below 500 µm.

For the biocompatibility and skin tests samples with different variants of infill were produced in order to investigate the influence of porous and closed structures on the wound dressing. The samples were produced in the following manner: The bioinks were filled into cartridges of 10 ml size and placed in extrusion unit of the setup. Machine code (G-Code) was prepared using the opensource Slice3r software. The printing was performed using conical nozzles with a diameter of 0.41 mm at the tip, a printing speed of 15 mm/s and a layer height of 0.3 mm, which translates into a volumetric speed of 1.56 mm^3^/s. In order to reach this flow a minimum pressure of 3.8 bar in the cartridge is needed, however with increasing ZnO amount the minimum pressure had to be increased to 4.8 bar due to an increasing viscosity of the bioink. In this way two different sets of samples were produced, that fit the respective testing method: For dermatological tests cylinders with a diameter of 10 mm and a height of 1.2 mm were produced. The infill was set to 100% in order to evaluate the effect of a closed structure of the wound dressing. Additionally, an infill of 50% was used to print samples consisting of a porous woodpile structure. This structure was designed in a way that no direct path from the top onto the sample is produced, as can be seen in Fig. 3A–F. This may possibly lead to a reduction of contamination by bacteria and still provide an open structure for oxygenation. For antibacterial tests, cubes with the dimensions of 1 mm × 1 mm × 1.2 mm were manufactured, also with different amounts of infill. For closed structures, again 100% was chosen, however for the porous samples the infill was reduced to 20%. After printing the samples were crosslinked in a 1 molar solution of calcium chloride for about 60 s and stored at −80 °C.

### Material characterization

#### Morphological analysis

The t-ZnO was visualized using the method of scanning electron microscopy (SEM). Firstly, they were attached to an aluminum stub with double-sided carbon tapes and then coated for 40 s with gold using the BAL-Tec SCP 050 Sputter Coater (Bal-Tec AG, Balzers, Liechtenstein). The samples were measured with the Phenom World XL (Thermo Fisher Scientific Inc., USA) scanning electron microscope at an acceleration voltage of 5 kV to 15 kV, a vacuum of 10 Pa and a spot size of 3.3 nm. The size of the tetrapodal arms as well as the overall particle diameters were statistically evaluated.

Additionally, SEM was performed on alginate/t-ZnO patches, that were lyophilized for 36 h.

Optical microscopy and fluorescence microscopy were performed on a Keyence BZ-X810 (Keyence, Neu-Isenburg, Germany). The BZ-X810’s software was used to create merged Z-stacks of the wound dressings and investigate their morphology over the whole height of the samples. The height of the samples was usually lower than 300 µm. Magnifications of 20x and 200x were used. The samples under investigation were hydrated alginate-based inks with t-ZnO contents of 0%, 5% and 15%.

#### Structural analysis

##### a. X-ray powder diffraction

Crystallinity of the t-ZnO powder was investigated with X-ray powder diffraction using a STOE STADI P diffractometer built-up with a transmission geometry and equipped with a Cu K_α_ anode in combination with a DECTRIS MYTHEN 1 K strip detector (STOE & Cie GmbH, Germany) in the range of 5° to 50° 2θ.

##### b. Micro Raman

Information on the orientation and bonding inside the crystal structure of the t-ZnO was investigated by Micro-Raman. The investigations were performed with a WITec system in a backscattering configuration at room temperature. The Nd:YAG laser power was less than 4 mW and the wavelength was λ_ex_ = 532 nm. For the spectrum 10 accumulations at an integration time of 0.5 s were taken. The samples investigated were pure alginate-based ink, alginate-based ink with 5% and 15% t-ZnO and pure t-ZnO.

#### Surface area

The specific surface area of a material can be determined by gas adsorption. A suitable method is based on the equation of Brunauer, Emmett and Teller (BET) with the assumption that the amount of adsorbed gas is proportional to the surface area of the sample. The BET theory was developed for the adsorption of N_2_ molecules but can also be used for other inert gases. An alternative method for determining the surface area is inverse gas chromatography (iGC) which can easily measure BET isotherms at room temperature using organic probe molecules.

The sample was filled into a 30 cm pre-silanized glass column to provide the stationary phase. Depending on the expected surface area of the sample different diameters of the glass columns could be used. The column was pre-conditioned with helium carrier gas to remove any volatile contamination. The specific surface area of tetrapodal zinc oxide was determined with heptane using the iGC-SEA (inverse gas chromatography surface energy analyzer, Surface Measurement Systems Ltd., UK). Heptane adsorption isotherms were measured at 303K and 0% relative humidity at a constant flow rate. The surface area (BET) was calculated from the corresponding heptane isotherms, within the partial pressure range of 5% to 35% P/P_0_ using the SEA analysis software (Surface Measurement Systems Ltd., UK).

#### Rheological analysis

Rheological measurements were performed on an MCR 702e Multidrive (Anton Paar, Austria). For all tests, a plate-plate configuration with a plate diameter of 8 mm was used. The linear-viscoelastic range was determined via an oscillatory amplitude sweep at constant frequency of 1 Hz. Alginate-based inks were loaded in the rheometer with a gap size of 0.6 mm. To measure the dynamic properties of the inks, flow curves were recorded. This was done by ramping the shear stress until the static yield point. Then, a shear rate control was used to limit the maximum shear rate to 3 1/s. When this shear rate was reached, the shear rate was decreased from 3 1/s at a rate of 0.6 1/s2 to a shear rate of 0. Over the duration of the experiment, shear stress and shear rate were recorded. From the data, the static and dynamic yield stress were calculated using the RheoPass software (Anton Paar, Austria).

### Protein adsorption

To assess the versatile absorption capability of t-ZnO, various proteins were used in the experiments as model substances. The disparity in the adsorption capacity on tetrapodal zinc oxide was evaluated. A varying loading efficiency, i.e., how much of the provided protein adsorbs to a fixed amount of t-ZnO, is expected to differ with the isoelectric point (IEP) of the protein. Therefore, proteins with different IEP were chosen as model substances. The proteins used are explained in more detail below. The protein concentrations were determined at a wavelength of 205 nm in a semi micro quartz cuvette (Hellma GmbH & Co. KG, Germany) with a Shimadzu UV-1280 (Shimadzu Corporation, Japan).

#### a. Bovine serum albumin

Bovine serum albumin (BSA), a protein of the blood plasma of the domestic cattle, consists of 583 amino acids and has a molecular mass of approximately 66 kDa. The IEP is at pH 4.9. The freeze-dried beige “powder” (Bovine serum albumin, art.no: A2153 , Merck KGaA, Germany) used for the adsorption experiments has a purity higher than 96% and is used as a reference substance in numerous biochemical experiments due to its inexpensive production [24].

#### b. Ovalbumin

Ovalbumin (OVA), the main component of chicken egg white, is a glycophosphoprotein consisting of 385 amino acids. With a molecular mass of about 44 kDa, it is sufficiently large and complex to be immunogenic. For this reason, it is often used as a model antigen for immunological research. Ovalbumin denatures at a temperature of 84.5°C and has an IEP at pH 4.5. The freeze dried beige “powder” used (Albumin from chicken egg white, art.no: A5503 Merck KGaA, Germany) has a minimum purity of 98% [25, 26].

#### c. Alpha-lactalbumin

Alpha-lactalbumin, a small, globular whey protein, consists of 123 amino acids and has a molecular mass of approximately 14 kDa. The IEP is at pH 4.4. The freeze dried white to beige powder used (Alpha-lactalbumin from bovine milk, art.no.: L5385, Merck KGaA, Germany) has a purity of at least 85%.

#### d. Avidin

Avidin, a component of chicken egg white, is a glycoprotein containing four identical subunits. The monomer consists of 128 amino acids. This results in a total molecular mass of approximately 66 kDa and an IEP at pH 10.5. The freeze-dried white to beige powder used (Avidin from egg white, art.no.: 189725, Merck KGaA, Germany) has a purity higher than 98%.

#### e. Lysozyme

Lysozyme, obtained from chicken eggs, consists of 129 amino acids and has a molecular mass of approximately 14 kDa. The IEP is at pH 11. The lysozyme used for the adsorption experiments (Lysozyme, art.no.: L7651, Merck KGaA, Germany) has a purity of at least 90%.

### Adsorption experiments

t-ZnO should be capable for being loaded with a protein because of their polarity [27]. The adsorption capacity of zinc oxide tetrapods was tested according to the monograph Ph. Eur.: “Hydrated aluminium hydroxide for adsorption” [28] with some modifications. The t-ZnO was weighted or pipetted according to the target concentration of 1 mg/ml, i.e., 2 mg zinc oxide tetrapods for a batch size of 2 ml. After the respective protein solution had been added to the samples with a target concentration of 200 µg/mL, the t-ZnO was incubated with the protein while being exposed to a 360° rotation at room temperature for 1 h. During this process the t-ZnO was kept in permanent contact with the dissolved protein. To perform the rotation, the adsorption wheel used was rotated at a speed of 20 rpm using a friabilator (ERWEKA GmbH, Germany). Both are depicted in Additional file 1**: Figure S1**. Finally, the suspensions were centrifuged at 10,000 g for 5 min at 25°C with the Centrifuge 5430 R. The protein content of the supernatant was analyzed at 205 nm with a calibration curve using a semi micro quartz cuvette. The protein content adsorbed at the surface of the samples was calculated as the difference between the used amount of the protein and the measured protein concentration of the supernatant.

### Antibacterial tests

For the antibacterial tests, *Staphylococcus (S.) aureus* (ATCC 29213) was used, as it is a typical wound pathogen with the potential for multi-resistance against antibiotics and was, therefore, chosen as a model microorganism for the evaluation of the antibacterial capabilities of the alginate-based wound patches. In preparation for the antibacterial tests, printed wound dressings were thawed for 15 min, before being submerged in a 1 m CaCl_2_ solution for 5 min for crosslinking. After submersion, gels were washed in distilled water for 5 min. The testing protocol was based on a method used in a previous study [9]. A volume of 100 µl of an overnight culture of bacteria were incubated with 10 ml tryptic soy broth (TSB; art.no: 22092, Sigma-Aldrich, USA) medium at 37 °C. Cell count was estimated by measuring the optical density at 600 nm after 1.5 h. Bacteria were diluted to a concentration of 1 × 10^5^ bacteria/ml in a solution consisting of 1% TSB medium in phosphate buffered saline (PBS; art.no.: L 1825 Bio & Sell, Germany). A volume of 36 µl of this solution were given directly onto each wound dressing. The wound dressings were incubated at 37 °C in a Petri dish containing moistened filter papers to prevent evaporation of growth medium. After 24 h, the wound dressings and the liquid pooled around them were transferred into 1 ml PBS and vortexed for 30 s. The number of surviving bacteria was determined by plating serial dilutions on Müller-Hinton agar (Bio-Rad, art.no. 64884, USA).

### Biocompatibility and skin tests

#### Incubation of ex vivo skin with the wound dressings

Human skin explants were obtained from plastic reduction surgeries after informed consent was obtained. The use of human skin for this study was approved by the Local Ethics Committee of the Medical Faculty, Kiel University, Germany (D 603/22) in accordance with the Declaration of Helsinki Principles guidelines. Ex vivo skin was washed with phosphate-buffered saline (PBS, art.no.: PBS-1A, Capricorn, Germany) and subcutaneous fat and tissue were removed carefully. Samples were incubated for 1 h at 37 °C in PBS/ 1% penicillin–streptomycin solution. Subsequently, skin was cut with a 12 mm biopsy punch in defined parts. Skin explants were placed in a 12 well plate in tissue culture inserts (pore size 0.4 µm, Sarstedt, Germany). The surrounding space was filled with 1 ml Dulbecco’s Modified Eagle Medium (DMEM, art.no: D5796, supplemented with 10% fetal calf serum, 2 mM L-glutamine, 10 µg/ml insulin and 10 ng/ml hydrocortisone, Sigma Aldrich, Germany). Skin explants were covered without or with wound dressings in different conditions (closed structure or open structure in t-ZnO-loaded concentrations with 0%, 5% or 15% respectively) and incubated (37 °C, 5% CO_2_) for 48 h.

#### Analysis of cytotoxicity

After incubation the surrounding medium was collected and potential cytotoxic effects were analyzed by the Cytotoxicity Detection Kit (LDH, Roche, Switzerland obtained from Sigma Aldrich, Germany, art.no: 4744926001) according to the manufacturer’s protocol. This procedure is based on the release of lactate dehydrogenase (LDH), a stable cytoplasmic enzyme released by damaged cells [29]. Released LDH was detected by an enzymatic reaction resulting in a formazan-class dye which was measured by absorbance at 492 nm. Skin without a wound dressing served as low control and treatment with the detergent Triton X-100 (concentration of 0.1%, art.no: X100-5ML, Sigma Aldrich, Germany) served as positive control since Triton X-100 damages the cells resulting in increased LDH release.

#### RNA isolation and real-time PCR

Skin explants were divided and one part was embedded for immunohistochemistry staining and one part was used for Trizol-based RNA isolation with Crystal RNAmagic according to the manufacturer’s protocol (Biolabproducts, Gödenstorf, Germany). After reverse transcription with oligo dT primer and PrimeScript reverse transcriptase (TaKaRa Bio,, art.no.: 2680B, Saint-Germain-en-Laye, France) real-time PCR was performed as recently described [30]. The following primer pairs were used: Filaggrin (FLG): 5`-GGC AAA TCC TGA AGA ATC CAG ATG-3`(forward primer and 5`-GGT AAA TTC TCT TTT CTG GTA GAC TC-3`(reverse primer); human beta-defensin-3 (hBD-3): 5`-TGT TTG CTT TGC TCT TCC TGT-3`(forward primer) and 5`-CGC CTC TGA CTC TGC AAT AA-3`(reverse primer); transglutaminase 1 (TGM-1): 5`-CCC TCA CCA ATG TCG TCT TC-3`(forward primer) and 5`-TCA CTG TTT CAT TGC CTC CA-3`(reverse primer). All quantifications were normalized to the housekeeping gene RPL38 (ribosomal protein L38) using the primer pair: 5`-TCA AGG ACT TCC TGC TCA CA-3` (forward primer) and 5`-AAA GGT ATC TGC TGC ATC GAA -3` (reverse primer).

#### Immunohistochemistry

Immunohistochemistry staining was performed as previously described [31]. As first antibody a polyclonal anti-Loricrin antibody (PA5-30583, Invitrogen, Karlsruhe, Germany) was used in a 1:500 dilution followed by biotinylated swine anti-rabbit IgG antibody (1:300; E0431, DakoCytomation, Glostrup, Denmark) and avidin/biotinylated enzyme complex (Vectastain ABC-AP staining-kit, Vector Laboratories, Peterborough, UK) and subsequently an alkaline phosphate substrate (Red AP, Vector Laboratories, Peterborough, UK). Slides were counterstained with hematoxylin (Merck, Darmstadt, Germany) and mounted with poly(butyl methacrylate-co-methyl methacrylate (Eukitt, O. Kindler, Freiburg, Germany).

## Results and discussion

The following segment shows the results obtained by preparing t-ZnO-laden alginate-based wound dressings in various modifications. Additionally, the protein binding capabilities of t-ZnO are presented and discussed.

### Material characterization

#### Morphology

**Fig. 2:**
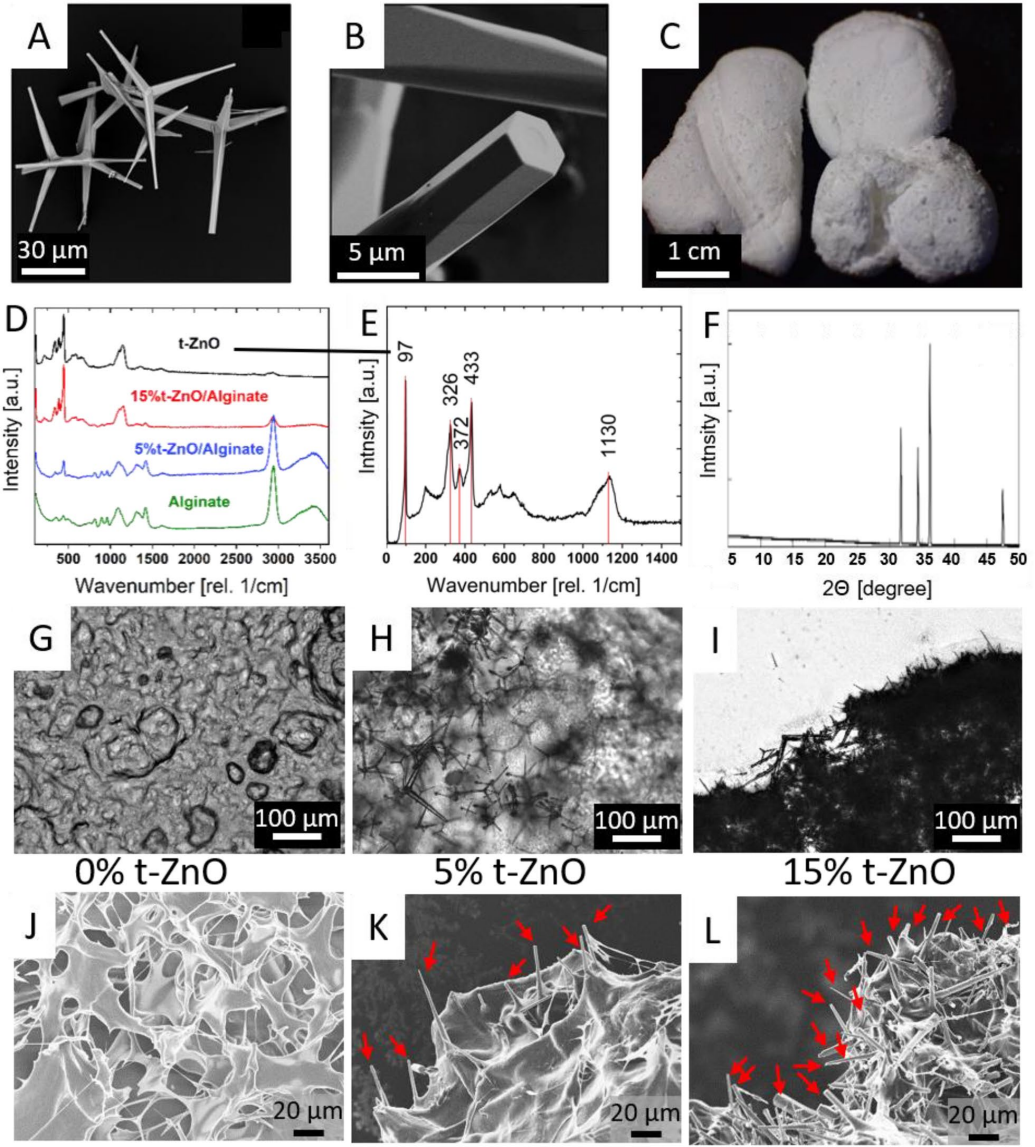
Morphological, structural and chemical analysis of the tetrapodal zinc oxide. **A** and **B** SEM micrograph of the tetrapods. The four arms protruding at fixed angles are visible in A, the hexagonal shape of each arm is visible in B. **C** A photograph of the t-ZnO powder. **D** Micro-Raman spectrum of alginate, zinc oxide and composites of 5% and 15% t-ZnO in alginate. **E** Enlarged micro-Raman spectrum of t-ZnO. Some characteristic peaks are marked with read lines and their corresponding wavenumber. **F** Diffractogram of the t-ZnO powder. The reflexes found indicate the crystallinity of the t-ZnO. **G**–**I** Optical micrographs of alginate-based inks with concentrations of 0%, 5% and 15% t-ZnO. **J**–**L** SEM micrographs of the alginate-based inks after lyophilization. The tetrapodal arms sticking out of the dried hydrogel can be clearly observed for K (5% t-ZnO) and L (15% t-ZnO)

The zinc oxide particles consist of four individual arms with a large aspect ratio and can be observed in the SEM micrographs of Fig. 2A and B. Each arm shows six facets, forming a hexagonal crystal. Statistical analysis of the particles shows that the average length of the arms is ~45 µm ± 20 µm and the overall particle size is ~80 µm ± 40 µm. The thickness of the arms can differ a lot, especially at their tips. Nevertheless, a thickness of approximately 200 nm up to 2 µm is most often found [18, 19]. In their diameter, the tips are a little smaller than human fibroblasts with approximately 10-15 µm yet too large to penetrate into the cells. As previously shown, t-ZnO has much lower effect on human fibroblasts than even commonly accepted for the cell-compatible ZnO nanoparticles [32–34]. When synthesized, the t-ZnO tends to form larger, fluffy agglomerates, which can be seen in Fig. 2C. Alginate-based wound dressings containing 0%, 5% and 15% ZnO were investigated with optical microscopy. The micrographs can be observed in Fig.  2G–I, with increasing t-ZnO concentration. In Fig. 2G, pure alginate-based ink can be seen with air bubbles and some surface roughness. The other two images show the added t-ZnO inside the alginate-based ink. The individual zinc oxide tetrapods can still be distinguished at a concentration of 5% in Fig. 2H. This is not the case in Fig. 2I, where the t-ZnO particles shadow each other. The high density of particles can be partially observed in fluorescence microscopy, where a UV-excitation and a blue filter is used to show the blue autofluorescence of t-ZnO. The images are taken as a z-stack and are depicted in Additional file 1: Figure S3. These images show a homogeneous distribution, which is difficult to achieve with complex shaped particles like t-ZnO. The high base viscosity of the alginate-based ink facilitates both the distribution and maintains the separation of the particles. Without this high base viscosity, the particles would sink down in the solution and get interlocked with each other in the process, forming agglomerates.

#### Crystal structure of t-ZnO

In order to investigate the degree of crystallinity of the t-ZnO, XRD measurements on the powdered material was performed. The main characteristic reflexes (100), (002) and (101) as well as (102) were found at the expected positions regarding the wurtzite type structure of zinc oxide [19]. The sharpness of the reflexes indicates a high crystalline quality.

#### Chemical analysis

In order to study the composition of the t-ZnO Raman scattering measurements via micro-Raman were performed. Major peaks were found at 97 cm^−1^, 326 cm^−1^, 272 cm^−^1, 433 cm^−1^ and 1130 cm^−1^ and they belong to the low-E_2_, E_2H_-E_2L_, A_1T_ high-E_2_ and 2 LO. The modes A_1_ and E_1_ are polar and split into transverse optical (TO) and longitudinal optical (LO) components [35]. The E_2_ modes are Raman active only and consist of low and high-frequency phonons (denominated as E_2L_ and E_2H_) [36]. The 2 LO peak is related to an optical overtone, i.e., a second-order peak [37]. The low-frequency mode of E_2_ in zinc oxide is associated with the vibration of the heavy Zn sub-lattice. Accordingly., in the E_2_ (high) mode only the oxygen atoms are involved. This mode is also characteristic of the wurtzite structure [38].

#### Rheological analysis

**Fig. 3:**
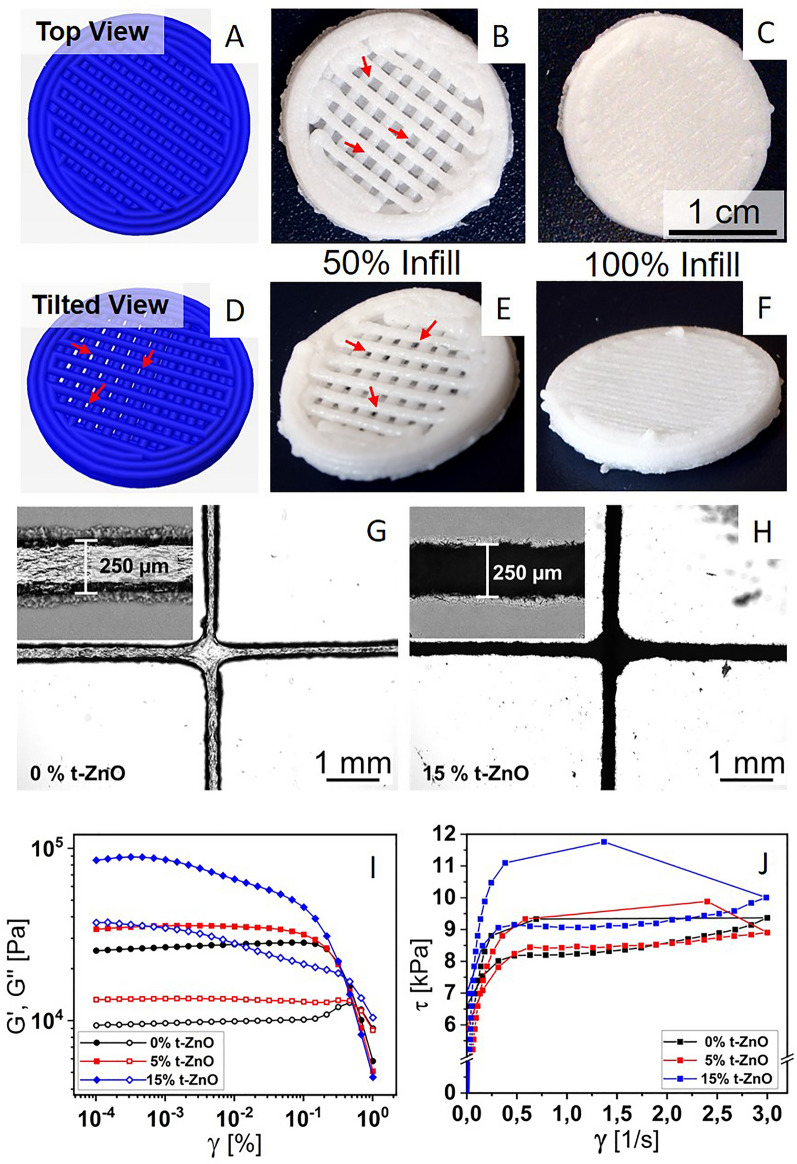
Bioprinting of the alginate-based inks. **A**–**F** Simulated and printed constructs. The pattern is picked in a way that no contaminants can enter from the top in a direct way. Still, open porosity, yet no direct path is implemented for the 50% filled constructs to retain oxygen permeability. **A** and **D** depict the simulated constructs generated in the slicing software from the respective G-Code. **A** represents the top view, where no direct path through the construct can be perceived. **D** shows the same constructs at a tilted angle where the open structure can be observed. **B** and **E** show the final printed constructs at a filling factor of 50% in the same viewing angles as **A** and **D**, showing both the blocked and the open pathways as stated before. **C** and **F** show the constructs with complete filling of 100%, with no easy pathways for oxygen or contaminants. **G** and **H** are optical micrographs depicting two crossing lines of printed alginate-based ink with 0% and 15% t-ZnO loading, respectively. The insets show a magnification of an individual line. The more jagged contour of the lines in H are caused by protruding t-ZnO arms. **I**: Graphs of an amplitude sweep for alginate-based inks with 0% to 15% t-ZnO. The moduli increase with the content of t-ZnO. **J**: Flow curves of alginate-based inks with 0% to 15% t-ZnO. Both static and dynamic yield stress do not change from 0 to 5% t-ZnO content. A significant increase in static and dynamic yield stress can be seen for 15% t-ZnO

Amplitude sweeps for all inks were performed to find the shear modulus and loss modulus in the linear viscoelastic region as well as the flow point, which is characterized by the intersection between the shear modulus (G’) and loss modulus (G’’) curves. The loss factor tan δ is the ratio between the loss modulus and the storage modulus. The results are shown in Fig. 3I and Additional file 1: Figure S2. In Fig. 3I the amplitude sweep for alginate-based bioinks with 0%, 5% and 15% t-ZnO content are shown. One can observe an increase in both the storage and loss moduli with increasing t-ZnO content. The storage modulus increases from 25 kPa to 34 kPa and finally 85 kPa for 0% up to 15% t-ZnO content. Likewise, the loss modulus increases from 9 kPa to 13 kPa and then 37 kPa. The loss factor, i.e., the ratio between storage modulus and loss modulus stays nearly constant as is shown in the Additional file 1: Figure S2.

The shape of the curves, the loss factor and the flow point stay nearly identical, even when adding more t-ZnO into the hydrogels, just the absolute value of the shear modulus and storage modulus changes. The increase in storage and loss moduli from 5% to 15% t-ZnO is approximately 3-fold, indicating that the modulus increases linearly with the concentration of t-ZnO. The increase from the pure alginate based bioink to the concentration 5% t-ZnO is 40%.

### 3D-printing

Simulations of the produced machine code are shown in Fig. 3A and D. It was used to manufacture porous structures with an infill of 50% from the alginate bioinks. The different viewing angles show, that the chosen printing pattern prevents direct paths through the sample, reducing the probability for contaminations reaching the wound. However, the structures porosity still allows an easy oxygen access at the bottom side of sample making it ideal for wound dressing. The printing results shown in Fig. 3B and E with a ZnO concentration of 15% coincide with the rheological data. The alginate ink exhibits high shape retention so even fine infill structures are printable. The individually printed lines of the infill pattern have a diameter of approximately 250 µm as it is shown in the Figs. 3G and H, however this highly depends on the hydration of the alginate. The microscope images were taken after a drying at room temperature leading to a shrinkage of the hydrogel. Crosslinking the alginate in calcium chloride solution on the other side leads to swelling of the samples. Nonetheless the individual lines remain separated from each other, since the crosslinking occurs mostly on the surface of the lines. This procedure guarantees, that the pathways through the printed structure still remain open even after the material swells. Additionally, the microscopy reveals that the tetrapod microparticles are not only inside of the printed bioink, but also stick out of the surface allowing direct contact with the wound to utilize to the ZnO’s anti-bacterial properties.

### Protein adsorption

**Fig. 4:**
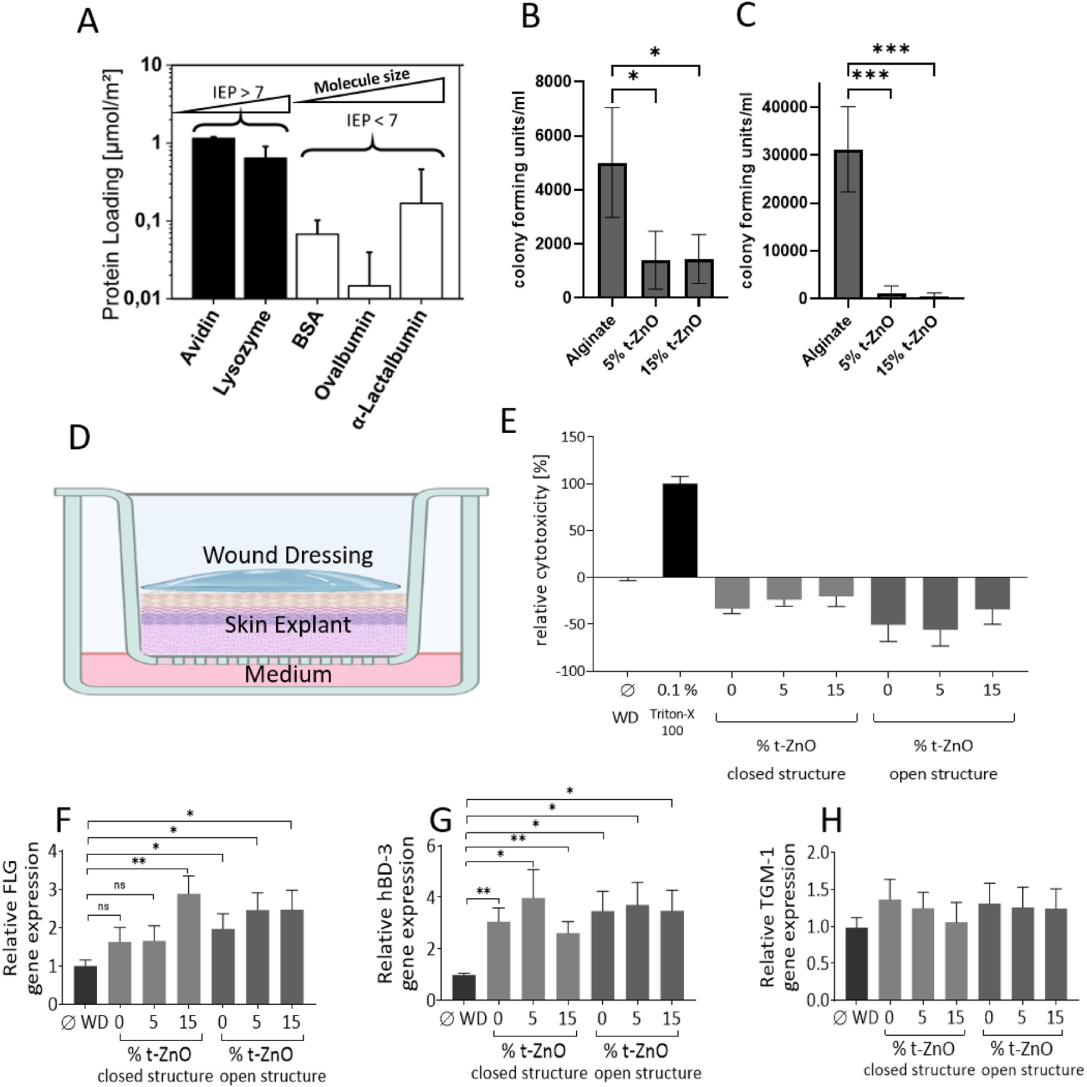
Protein adsorption and cell reactions of alginate-based t-ZnO bioinks. **A**: Protein loading efficiency plot of five different proteins on t-ZnO. The first two, filled bars show proteins with an IEP above 10 and high loading efficiency. In comparison the unfilled bars represent proteins with an IEP below 6. These exhibit a much lower loading efficiency than the first two proteins, hinting at a relation between loading efficiency and IEP. **B**-**C**: Antibacterial activity of t-ZnO-laden alginate hydrogels against *S. aureus*. The number of surviving bacteria of *S. aureus* cultured on t-ZnO-laden alginate hydrogels with different structures and containing differing amounts of ZnO (n = 4, *p ≤ 0.05, ***p ≤ 0.0005). In these experiments, **B** represents a closed structure, **C** represents an open lattice structure. **D-H**: Ex vivo skin explants were covered with or without wound dressings (WD) containing different t-ZnO concentrations (0; 5 or 15% respectively) and incubated for 48 h. Skin explants without WD served as a control. **D** shows the Schematic experimental setup (created in biorender.com). **E**: Lactate dehydrogenase (LDH)-release was measured in the surrounding supernatant. Skin explant in medium with Triton-X 100 0.1% served as high control. Measurement shows cytotoxicity (%) relative to Triton-X 100. **F**–**H**: After RNA isolation and reverse transcription from the remaining parts of skin explants real-time PCR analysis of FLG, hBD-3 and TGM-1 was performed. Shown are means ± s.e.m. of three independent ex vivo skin explants from different patients consisting of two to four stimulations (*p < 0.05; **p < 0.01; ns = not significant; paired t test)

The concentration of API (e.g., a protein) is the decisive factor in the efficacy of wound patch treatment. Thus, the protein adsorption capacity on the surface of t-ZnO was evaluated. The most reasonable approach to check for the loading efficiency is to calculate the number of molecules per surface area. This then excludes the proteins molecular mass and therefore it’s approximate size. This is potentially problematic, because the size of the molecules has to be considered in the evaluation. The protein loading was calculated by using the surface area of the tetrapods. The surface area of the tetrapods as determined by SEA is rather low with 0,373 m^2^/g. The reason is that t-ZnO is not a nanomaterial, i.e., it has a much lower surface-to-volume ratio than, e.g., ZnO nanoparticles. The results of the protein adsorption tests utilizing several model proteins of different characteristics are depicted in Fig. 4A. A clear difference can between the proteins with an alkaline IEP above pH 7 and those with acidic IEP below pH 7 can be observed. Avidin and Lysozyme both have an IEP above 10, rendering them positively charged at neutral pH. From the logarithmic plot, it can be seen that the loading efficiency with these two proteins is almost 10 times higher than those with an IEP below 7. Bovine serum albumin, ovalbumin and α-Lactalbumin all are negatively charged under neutral conditions. This indicates that the charge of the molecule in solution is a decisive factor for the loading efficiency of the active ingredient on t-ZnO. Zinc oxide tends to exhibit oxygen vacancies on its surface even under ambient conditions and these are negatively charged [39–41]. Therefore, the ZnO particles exhibit a slightly negative surface charge. To attach a molecule to the surface of a ZnO particle, it should match the charge present on the surface. This is very likely the reason why the more positively charged proteins are adsorbed to the surface to a higher extent than the negatively charged ones. This effect is not superimposed significantly by the size of the molecules. Avidin, for example, is with 66 kDa the largest molecule, yet at the same time can be loaded most efficiently out of all the proteins tested. In comparison, α-Lactalbumin (14 kDa), Ovalbumin (44 kDa) and BSA (66 kDa) increase in size, yet no clear trend towards their loading efficiency can be observed. Thus, the IEP seems to have a much larger influence on the protein adsorption than the molecule’s size. SEM was performed on the t-ZnO after protein loading. The micrographs are shown in Additional file 1: Figure S4. Most of the proteins formed smooth films on the t-ZnO. One exception is Avidin, which formed agglomerations on the surface of the t-ZnO. This may be an expression of the tendency of the protein to bind to itself in combination with the size of the molecule. A tendency of the protein to bind to itself could increase the loading efficiency.

Considering t-ZnO as a platform for carrying API, the charges in solution have to be considered primarily for the loading to be efficient. However, the overall efficacy of t-ZnO-delivered API is high, because of the direct contact between the surface of the alginate-based wound dressing and the desired tissue. The direct contact alleviates the need for diffusion, so the interaction is local and strong. Therefore, a much lower loading of t-ZnO in the bioink is necessary than for bulk release from nanoparticle-laden hydrogels.

### Antibacterial tests

Bacteria, including facultative pathogens, are commonly found on the human skin as commensals. While they usually cause no problems on healthy skin, damage to the mechanical barrier posed by the epidermis may lead to bacterial invasion and cause wound infection [42]. This can result in further tissue damage and may even lead to chronic wound formation. It is therefore necessary to keep the wound tissue free from bacteria to prevent further damage and ensure proper healing. In addition to common antibiotics, antibacterial wound dressings are a possibility to prevent bacterial wound infection. The antibacterial effect of t-ZnO-laden alginate wound dressings was evaluated by measuring the growth of *S. aureus*, a common pathogen often associated with wound infections [43].

The antibacterial tests showed that alginate wound dressings with an open lattice structure containing 5% t-ZnO were able to effectively reduce the growth of *S. aureus* after 24 h of incubation by a factor of 10 in comparison to the pristine alginate gels (Fig. 4B, C). Incubation on open lattice wound dressings containing 15% t-ZnO displayed an even greater reduction in the growth of *S. aureus*, resulting in a reduction of surviving bacteria by a magnitude of 10^2^. Similar results could be obtained for closed lattice wound dressings containing 5% and 15% t-ZnO respectively, although the growth reduction was less pronounced. Both types of t-ZnO-laden gels exhibited a three- to fourfold growth reduction. It has to be noted that growth of *S. aureus* on the closed lattice wound dressings seemed to be inferior in comparison to the open lattice ones, even without the addition of t-ZnO. In experiments not shown here, a not fully crosslinked alginate wound dressing showed no significant antibacterial effect, even at 15% of t-ZnO content. In previous studies it was proposed that the antibacterial activity of t-ZnO is due to the release of either Zn^2+^ ions or the generation of reactive oxygen species (ROS) [9, 44]. It may be that the open lattice structure supports the release of Zn^2+^ ions or ROS due to a larger surface area in contact with the growth medium. If the alginate is not crosslinked, it can capture the Zn^2+^ ions that cannot have an antibacterial effect then. Therefore, a fully crosslinked t-ZnO containing open lattice wound dressing is the most favorable to exhibit the best antibacterial activity.

The results of this study show that t-ZnO laden alginate wound dressings possess antibacterial activity against *S. aureus* and therefore may prevent wound infection by this pathogen and maybe other gram-positive bacteria. Previous studies showed that t-ZnO possesses antibacterial properties against a range of pathogenic bacteria [44]. Therefore, the applicability of t-ZnO laden alginate wound dressings may also extend to wounds infected with other kinds of bacteria.

### Biocompatibility and skin tests

To analyze the influence of the wound dressings on the skin integrity, we applied the wound dressings containing 0%, 5% and 15% t-ZnO on *ex vivo* skin explants for 48 h. Skin explants without wound dressing served as a control. First, we evaluated potential cytotoxic effects of the wound dressings by analyzing the release of lactate dehydrogenase (LDH). As a result, we observed that the LDH release of the skin explants with all wound dressings was lower than the LDH release of control skin without wound dressing indicating no negative influence of the wound dressings on skin integrity (Fig. 4E).

To further analyze and visualize the condition of the skin barrier after application of the various wound dressings, we performed immunostaining of the differentiation marker loricrin. Loricrin is an abundant member of the cornified envelope of the skin and enhances the protective cutaneous barrier [45]. No marked change of loricrin expression was observed in the wound dressing-treated skin explants (Additional file 1: Figure S5). In addition, the overall morphology of the wound dressing-treated skin explants revealed no morphological signs of damage. The only obvious change in the wound dressing-treated skin as compared to the control skin was a slight swelling of the uppermost layers of the stratum corneum which was also seen in the skin treated with 0% t-ZnO wound dressings. This swelling of the stratum corneum was likely mediated by the occlusive effect of the wound dressings.

To further analyze the potential influence of the wound dressings on the skin barrier, we determined the gene expression of filaggrin and transglutaminase 1, two crucial factors of the skin barrier. Filaggrin (FLG) is a structural protein of the stratum corneum and its degradation products provide amino acid metabolites that act as natural moisturizing factors in the stratum corneum. FLG loss-of-function mutations are associated with the skin disorders ichthyosis vulgaris and atopic dermatitis [46]. Transglutaminase 1 (TGM1) is an enzyme responsible for cross-linking epidermal proteins during formation of the stratum corneum and is also involved in the proliferation of keratinocytes [47, 48]. TGM1 knockout mice died a few days after birth due to impaired skin barrier function and TGM1 mutations are associated with a form of the inherited skin disorder lamellar ichthyosis [49, 50]. As shown in Fig. 4F–H, application of the wound dressings for 48 h on *ex vivo* skin had no negative influence of TGM1 gene expression whereas filaggrin gene expression was even slightly induced by the various wound dressings. In addition, we observed that gene expression of the antimicrobial peptide human beta-defensin (hBD)-3 was slightly induced by the wound dressing [51, 52]. This suggests that the wound dressings may even strengthen skin defense, a hypothesis that needs to be investigated in further studies.

## Conclusions

In summary, we have designed, bioprinted and investigated a novel platform based on sodium alginate and t-ZnO for a patient-specific protein administration in wound care. The t-ZnO particles were used as the main active ingredient and were therefore screened for their capability to bind proteins to their surface. It was found, that generally, proteins with an isoelectric point above 7 showed a much higher tendency to bind to the t-ZnO surface. Since many cytokines and growth factors can potentially be used as the active pharmaceutical ingredient in such a wound patch, binding them to the surface of the complex-shaped t-ZnO particles is very beneficial. It was found that the t-ZnO sticks out of the printed alginate filaments and comes in direct contact with its surrounding tissue. Printing and investigating t-ZnO-laden alginate wound dressings in concentrations from 0 to 15% revealed that already at a concentration of 5% they show a strong anti-bacterial effect against *S. aureus*, yet exhibited no significant negative effect towards human skin. From two different constructs, open and closed structured, it was found that the open structure was much more anti-bacterial than the closed structure, while neither exhibited any obvious adverse effect towards human skin.

In total, we believe that this system may be used to investigate the effects of surface-bound proteins on the t-ZnO towards wounds, while generally providing many of the necessary factors for wound healing like oxygenation, hydration and an anti-bacterial effect.

### Supplementary Information

**Additional file 1**. Additional information regarding fluorescence microscopy, rheology and others.

## Data Availability

The datasets used and analyzed during the current study are available from the corresponding author on reasonable request.
