# Correction: 3D-printed wound dressing platform for protein administration based on alginate and zinc oxide tetrapods

**DOI:** 10.1186/s40580-024-00433-6

**Published:** 2024-07-08

**Authors:** Philipp Schadte, Franziska Rademacher, Gerrit Andresen, Marie Hellfritzsch, Haoyi Qiu, Gregor Maschkowitz, Regine Gläser, Nina Heinemann, Daniel Drücke, Helmut Fickenscher, Regina Scherließ, Jürgen Harder, Rainer Adelung, Leonard Siebert

**Affiliations:** 1 Department for Material Science, Functional Nanomaterials, Kiel University, Kiel, Germany; 2 Department of Dermatology, Kiel University and University Medical Center Schleswig-Holstein, Kiel, Germany; 3 Institute for Infection Medicine, Kiel University and University Medical Center Schleswig-Holstein, Kiel, Germany; 4 Department of Pharmaceutics and Biopharmaceutics, Kiel University, Kiel, Germany; 5 Department of Reconstructive Surgery, Kiel University and University Medical Center Schleswig-Holstein, Kiel, Germany; 6 Kiel Nano, Surface and Interface Science - KiNSIS, Kiel University, Kiel, Germany

**Correction to: Nano Convergence (2024) 10:53**.

10.1186/s40580-023-00401-6.

Following publication of the original article [1], the author identified an error in Fig. 3. The scale bar value should be “1 cm” instead of “1 mm” in panel C of the figure. The revised Fig. 3 has given below.

**Fig. 3:**
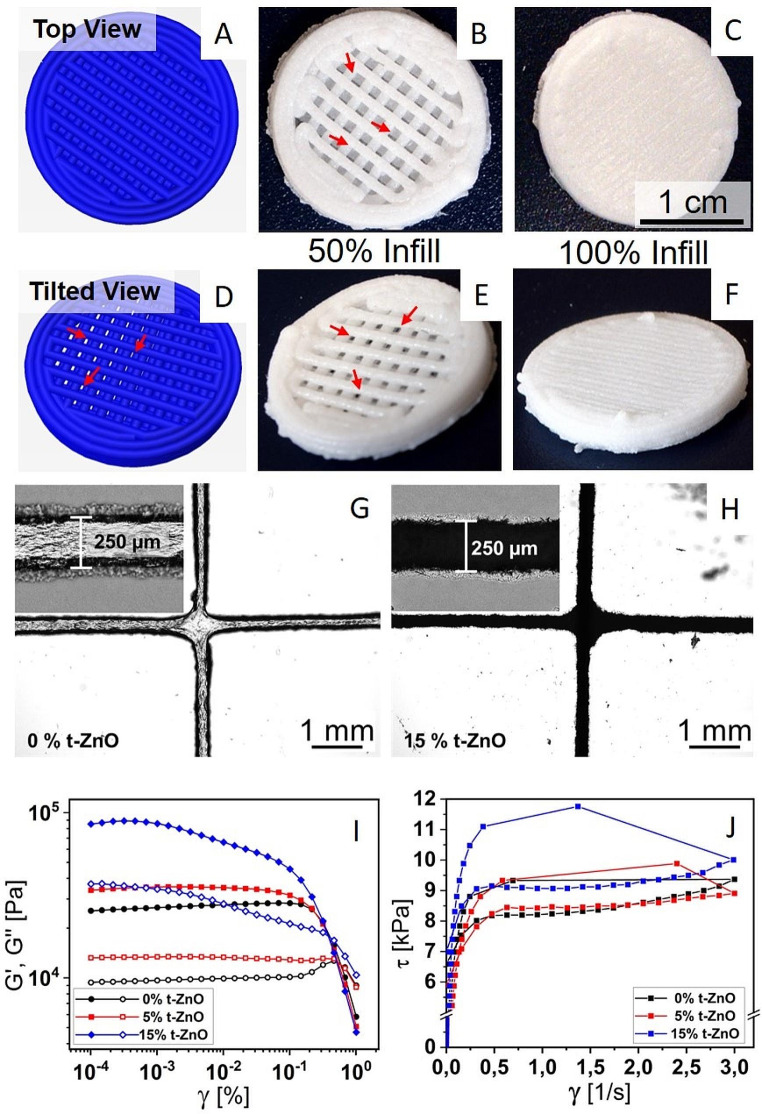
Bioprinting of the alginate-based inks. **A–F** Simulated and printed constructs. The pattern is picked in a way that no contaminants can enter from the top in a direct way. Still, open porosity, yet no direct path is implemented for the 50% filled constructs to retain oxygen permeability. **A** and **D** depict the simulated constructs generated in the slicing software from the respective G-Code. **A** represents the top view, where no direct path through the construct can be perceived. **D** shows the same constructs at a tilted angle where the open structure can be observed. **B** and **E** show the final printed constructs at a filling factor of 50% in the same viewing angles as **A** and **D**, showing both the blocked and the open pathways as stated before. **C** and **F** show the constructs with complete filling of 100%, with no easy pathways for oxygen or contaminants. **G** and **H** are optical micrographs depicting two crossing lines of printed alginate-based ink with 0% and 15% t-ZnO loading, respectively. The insets show a magnification of an individual line. The more jagged contour of the lines in H are caused by protruding t-ZnO arms. **I**: Graphs of an amplitude sweep for alginate-based inks with 0% to 15% t-ZnO. The moduli increase with the content of t-ZnO. **J**: Flow curves of alginate-based inks with 0% to 15% t-ZnO. Both static and dynamic yield stress do not change from 0 to 5% t-ZnO content. A significant increase in static and dynamic yield stress can be seen for 15% t-ZnO

The original article has been corrected.

