# Supplementary material for: 3D-printed wound dressing platform for protein administration based on alginate and zinc oxide tetrapods
Source: Nano Converg. 2023 Nov 16;10:53. doi: 10.1186/s40580-023-00401-6 (PMC10654273; doi:10.1186/s40580-023-00401-6)
Supplement: Supplementary file 1 — Additional file 1. Additional information regarding fluorescence microscopy, rheology and others. [file 40580_2023_401_MOESM1_ESM.docx]

**Supporting Information**

**to**

**Zinc Oxide Tetrapod-based Bioink as platform for protein administration and Antibacterial Wound Dressings**

Philipp Schadte^1^, Franziska Rademacher², Gerrit Andresen^3^ Marie Hellfritzsch^4^, Haoyi Qiu^1^, Gregor Maschkowitz^3^, Regine Gläser^2^, Nina Heinemann^2^, Daniel Drücke^5^, Helmut Fickenscher^3^, Regina Scherließ^4,6^, Jürgen Harder^4^, Rainer Adelung^1,6,*^, Leonard Siebert^1,6^

1. Functional Nanomaterials, Department for Material Science, Kiel University, Kiel, Germany.
2. Department of Dermatology, Kiel University, Kiel, Germany.
3. Institute for Infection Medicine, Kiel University and University Medical Center Schleswig-Holstein, Kiel, Germany.
4. Department of Pharmaceutics and Biopharmaceutics, Kiel University, Kiel, Germany.
5. Department of Reconstructive Surgery, University Medical Center Schleswig-Holstein, Kiel, Germany.
6. Kiel Nano, Surface and Interface Science - KiNSIS, Kiel University, Kiel, Germany.

- Corresponding author: Prof. Dr. Rainer Adelung

Tel.: +49 431 880 6116

E-mail address: ra@tf.uni-kiel.de

**Supporting Figure 1.**


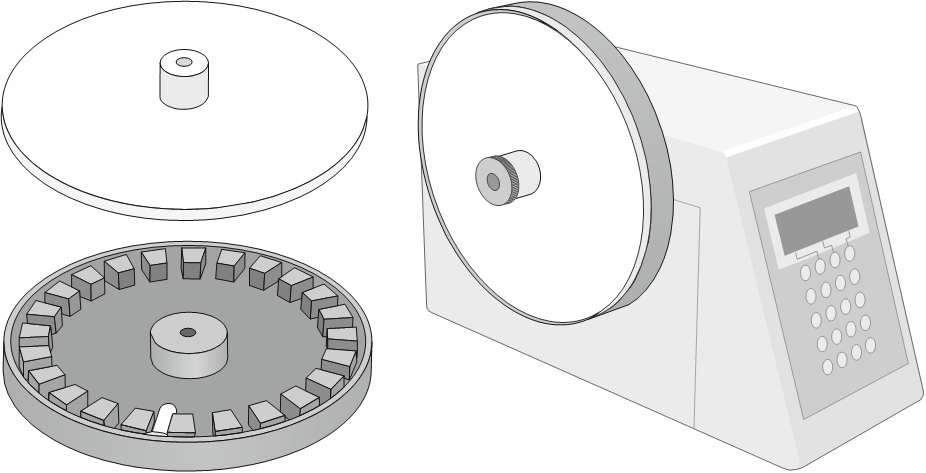


**Sup. Fig. 1.** Schematic picture of the adsorption wheel (left) in combination with the friabilator (right) used for the Adsorption Experiments. The Equipment was used to deposit the proteins on the t-ZnO particles.

**Supporting Figure 2.**

**
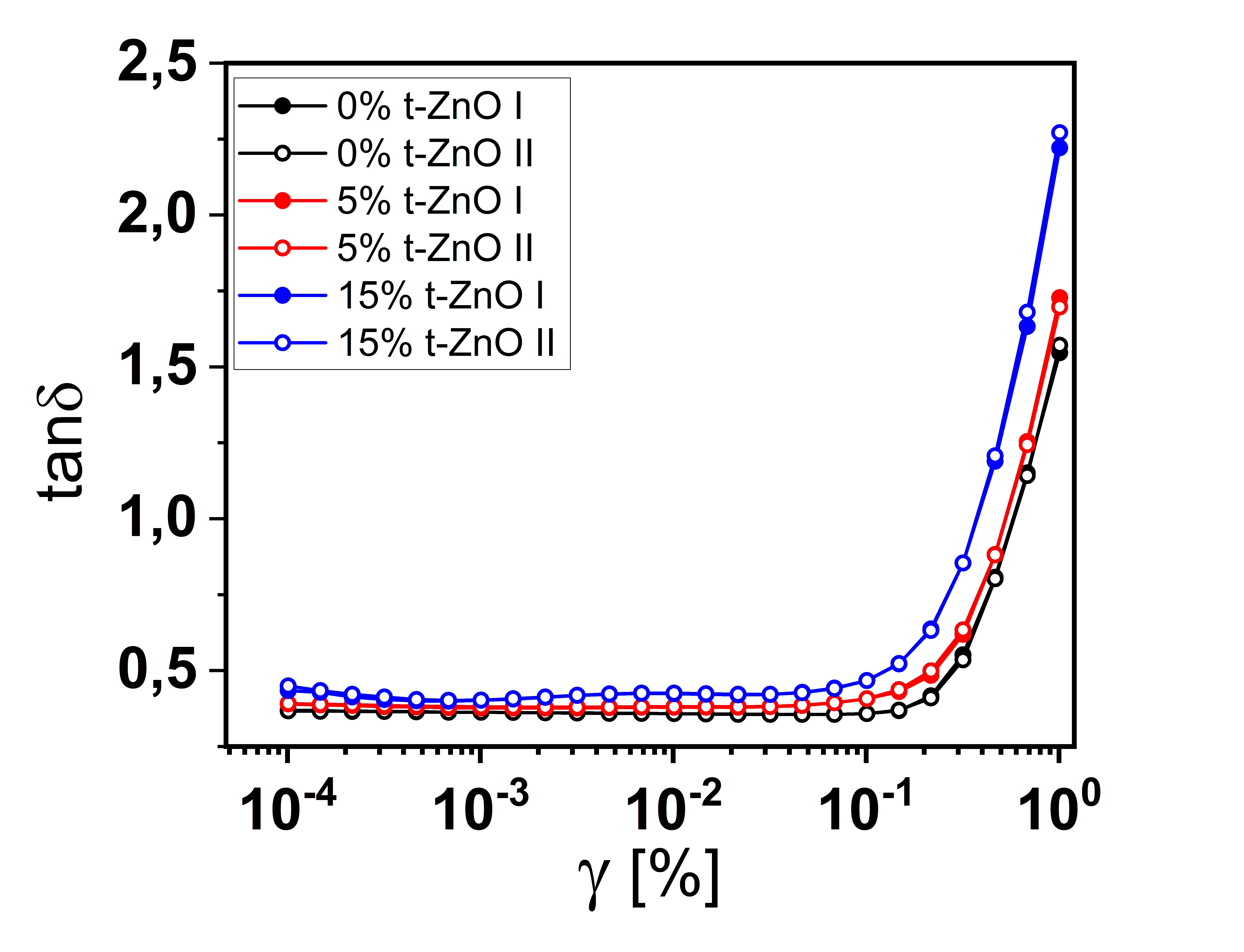
**

**Sup. Fig. 2.** Loss Factor tan δ of the amplitude sweeps of alginate-based bioinks at 0%, 5% and 15% t-ZnO content. Although the particle concentration is increased the loss factor stays constant for the investigated shear rates.

**Supporting Figure 3.**


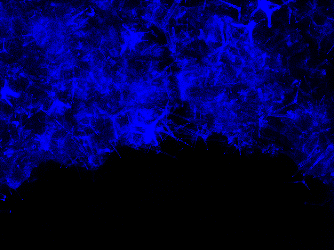

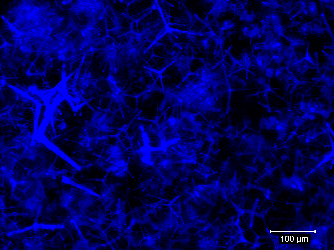


**Sup. Fig. 3.** Fluorescence Microscopy Images of t-ZnO in alginate hydrogel. Due to an UV-excitation and a blue filter the tetrapods could be observed inside the alginate. The images illustrate the homogeneous and at the same time dense distribution of particles in the bioink, which is kept stable by the high base viscosity.

**Supporting Figure 4.**


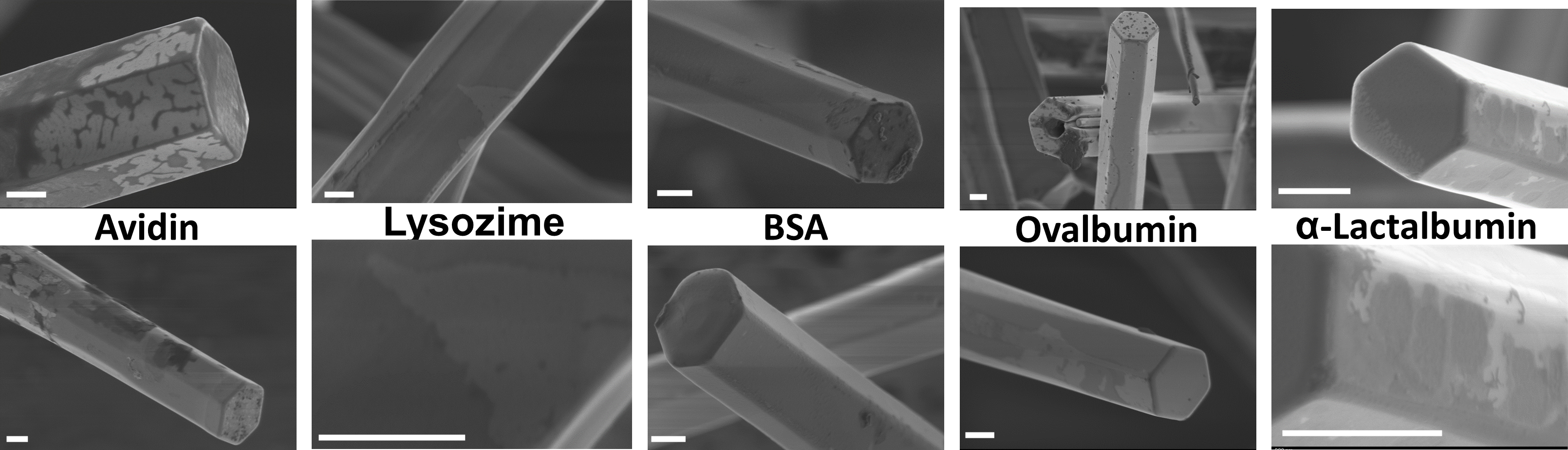


**Sup. Fig. 4.** SEM Images of t-ZnO loaded with various proteins (scale bar: 1 µm). The proteins form films on the surfaces of the tetrapod shown by the different shades of grey.

**Supporting Figure 5.**


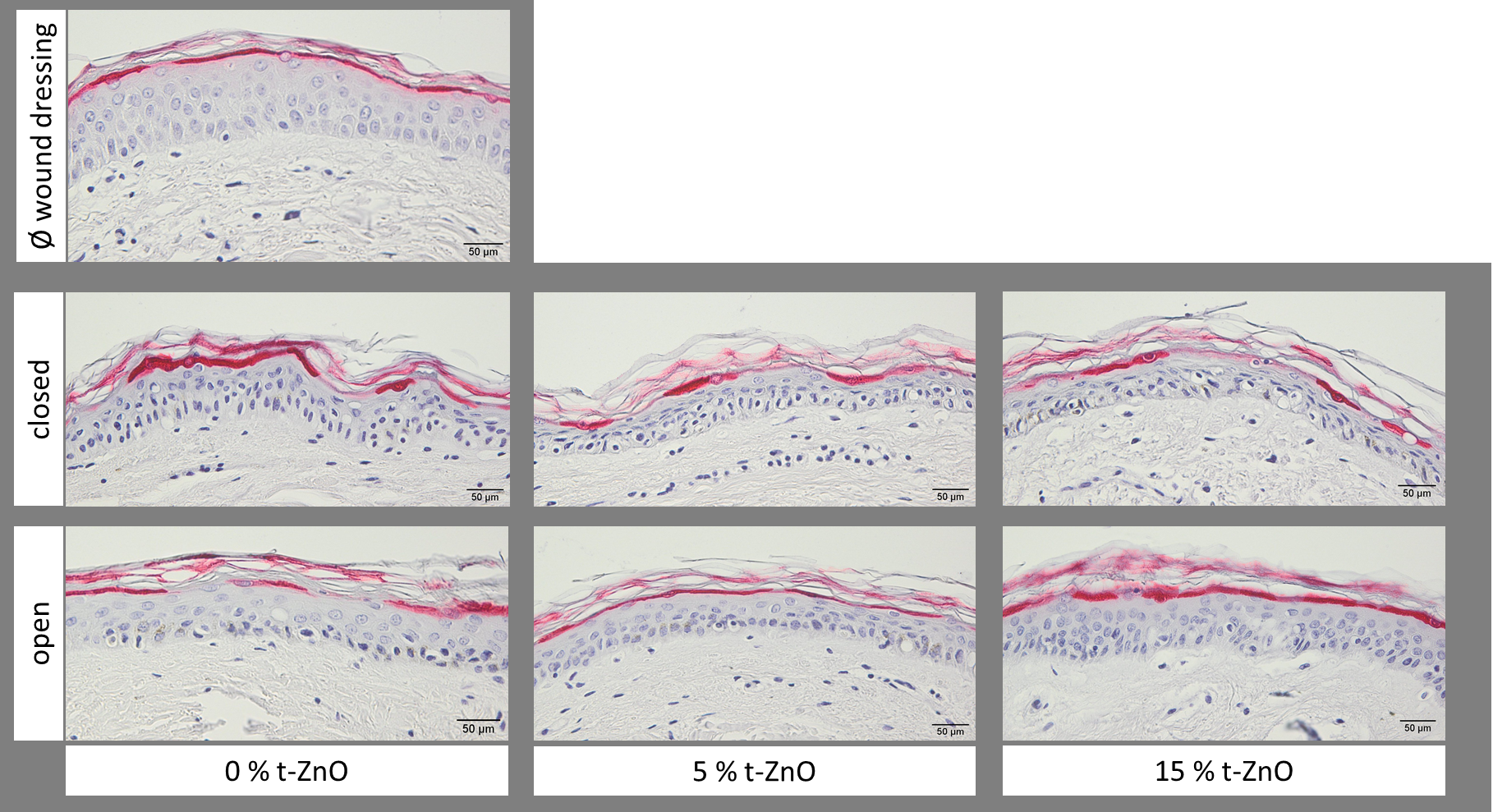
 **Sup. Fig. 5.** Skin explants were partly embedded in paraffin and immunohistochemistry staining of loricrin was performed in order to visualize the condition of the skin barrier after the application of the wound dressings. None of the wound dressing variations showed a negative influence on the skin, however a slight swelling of the stratum corneum was observed compared to the control skin.
